# Inhibiting Tyrosine Kinase 2 Ameliorates Antiphospholipid Syndrome Nephropathy

**DOI:** 10.1155/mi/5568822

**Published:** 2024-12-24

**Authors:** Kuo-Tung Tang, Yu-Sin Chen, Tzu-Ting Chen, Ya-Hsuan Chao, Shu-Ping Kung, Der-Yuan Chen, Chi-Chien Lin

**Affiliations:** ^1^Division of Allergy, Immunology and Rheumatology, Taichung Veterans General Hospital, Taichung, Taiwan; ^2^School of Medicine, National Yang Ming Chiao Tung University, Taipei, Taiwan; ^3^Program in Translational Medicine, National Chung Hsing University, Taichung, Taiwan; ^4^Graduate Institute of Biotechnology, National Chung Hsing University, Taichung, Taiwan; ^5^Institute of Biomedical Science, The iEGG and Animal Biotechnology Center, National Chung-Hsing University, Taichung, Taiwan; ^6^Institute of Bioinformatics and Structural Biology and Department of Medical Science, National Tsing Hua University, Hsinchu, Taiwan; ^7^Division of Chest Medicine, Department of Internal Medicine, Changhua Christian Hospital, Changhua, Taiwan; ^8^Rheumatology and Immunology Center, China Medical University Hospital, Taichung, Taiwan; ^9^College of Medicine, China Medical University, Taichung, Taiwan; ^10^College of Medicine, National Chung Hsing University, Taichung, Taiwan; ^11^Department of Medical Research, Taichung Veterans General Hospital, Taichung, Taiwan; ^12^Rong Hsing Research Center for Translational Medicine, National Chung Hsing University, Taichung 402, Taiwan; ^13^Department of Medical Research, China Medical University Hospital, Taichung, Taiwan; ^14^Department of Pharmacology, College of Medicine, Kaohsiung Medical University, Kaohsiung, Taiwan

**Keywords:** antiphospholipid antibody syndrome, nephropathy, thrombotic microangiopathy, type I interferon, tyrosine kinase 2

## Abstract

**Objective:** Antiphospholipid antibody syndrome (APS) is an autoimmune disease characterized by the presence of *β*2-glycoprotein I (*β*2-GPI)-targeting antiphospholipid antibodies (aPLs) and vascular thrombosis or obstetrical complications. One of its severe manifestations is nephropathy.

**Methods:** To examine the role of type I interferon (IFN) and therapeutic potential of tyrosine kinase 2 (Tyk2) inhibition, we administered BMS-986202, a novel Tyk2 inhibitor, in a mouse model of APS nephropathy. We administered BMS-986202 to BALB/c mice at a dose of 2 mg/kg. Biochemical and histological characteristics of APS nephropathy were then determined. The type I IFN signature in the kidney was also evaluated by real-time polymerase chain reaction (PCR).

**Results:** The Tyk2 inhibitor reversed the elevation of blood urea nitrogen (BUN) and microalbuminuria in the murine model of APS nephropathy. In addition, the Tyk2 inhibitor reversed the pathological vascular changes in the kidney as judged in electron microscopy (EM), and fibrin and C3 deposition as revealed in immunohistochemistry (IHC). An increased expression levels of IFN signature (IFN regulatory factor 7 (IRF7) and Mx1) in the kidneys of APS mice were found. Tyk2 inhibition reversed such an upregulation.

**Conclusion:** Our results demonstrated the key role of type I IFN in the pathogenesis of APS nephropathy. Furthermore, the therapeutic efficacy of Tyk2 inhibition was demonstrated in a murine model of APS nephropathy. Our results could provide a new treatment strategy for this debilitating disease.

## 1. Introduction

Antiphospholipid antibody syndrome (APS) is an autoimmune disease which is characterized by antiphospholipid antibodies (aPLs) [1] and vascular thrombosis or obstetrical complications. The main target antigen in APS is *β*2-glycoprotein I (*β*2-GPI) [2], a plasma glycoprotein participating in coagulation and complement regulation [3]. aPLs bind to the cell membrane through *β*2-GPI, and subsequently elicit signal transduction intracellularly [1], resulting in activating the complement [4–6] and the coagulation cascade. The autoimmunity towards *β*2-GPI is speculated to play a key role in the pathogenesis of APS.

Among the severe manifestations of APS, nephropathy is an important one. One third of APS patients develop nephropathy as revealed in biopsy [7–9]. Such nephropathy is histologically characterized by thrombotic microangiopathy and chronic vascular lesions such as fibrous intimal hyperplasia of interlobular arteries, fibrous occlusions, recanalized thrombi in arteries and arterioles, et cetera. The end result is acute kidney injury, hypertension, hematuria, proteinuria, and even chronic kidney dysfunction. In those patients undergoing renal transplantation; furthermore, the presence of aPL in the blood is associated with a lower survival rate of renal graft [10–12]. The underlying mechanism of APS nephropathy remains unclear. Seshan et al. [13] administered human and mouse aPL to mice and produced a murine model of APS in nephropathy. Their results implied the contributory role of tissue factor and complement activation in the pathogenesis of APS nephropathy [13]. To date, the treatment of APS relies on the use of antiplatelet and anticoagulants. No standard treatment for APS nephropathy has been established.

In recent decades, a contributory role of type I interferon (IFN) in the pathogenesis of autoimmune diseases like APS has been suggested [14]. The Janus kinase (JAK) family is crucial in the signal transduction of a myriad of cytokine receptors and its inhibition revolutionized the treatment for autoimmune diseases [15]. Tyrosine kinase 2 (Tyk2) is one of the JAK family members, through which interleukin (IL)-12, IL-23, and IFN-*α* exerts the downstream effects. Deucravacitinib (BMS-986165), a Tyk2 inhibitor that binds to the JAK homology 2 (JH2) domain, has been approved for treating psoriasis [16] and reported to be effective in the treatment of systemic lupus erythematosus (SLE) [17]. To be noted, APS is frequently accompanied with SLE. Since the JAK family share a similar structure of the JAK homology 1 (JH1) domain, a cross reaction against other JAK happens and may lead to unwanted side effects. BMS-986202 is a novel Tyk2 inhibitor that binds to the JH2 domain. It is expected to more selective, with greater potency and less side effects [18]. We hypothesized that type I IFN is crucial in the generation of APS nephropathy. In addition, we inhibited downstream Tyk2 signaling with BMS-986202 in a murine model of APS nephropathy to examine the therapeutic potential.

## 2. Materials and Methods

### 2.1. Animals

Six-week-old female BALB/c mice were obtained from the National Laboratory Animal Center (Taipei, Taiwan). Free access to food and water was kept. Their environment was maintained at 22 ± 2°C and 60% ± 10% humidity. Assuming the difference in renal function (blood urea nitrogen (BUN)) between treated and control mice being 20 mg/mL [13], the within-group standard deviation (SD) being 10 mg/mL, power of 0.8, and the type I error of 0.05, five mice are required for both groups to be able to reject the null hypothesis. Finally, six mice were assigned to each experimental group. All animal procedures were conducted according to institutional guidelines and approved by the Institutional Animal Care and Utilization Committee of National Chung Hsing University, Taiwan (approval protocol no. NCHU-IACUC-110-127).

### 2.2. Establishment of a Murine Model of APS Nephropathy

Our experimental procedures were modified from that conducted by Seshan et al. [13]. Immunization with *β*2-GPI (20 μg/mouse; Prospec, Rohovot, Israel) in the complete Freund's adjuvant (Chondrex, Redmond, WA) was performed in mice. They underwent booster immunization (*β*2-GPI of 20 μg/mouse in complete Freund's adjuvant) at day 21. Their blood samples were obtained from the orbital sinus on day 42. Urine samples were collected at the same time. After CO_2_ asphyxiation, their kidneys were then retrieved.

### 2.3. Measurement of the Anti-*β*2-GPI IgG Antibody

We incubated the whole blood sample obtained from each mouse for 1 h at 4°C, which was followed by centrifugation at 2500 rpm. The serum samples were then collected, and diluted in tris-buffered saline (1:100; pH 8.0) which contained 1% BSA and 0.5% Tween-20. Anti-*β*2GPI IgG concentrations in the serum were measured by an in-house ELISA. In brief, we added human *β*2-GPI (Prospec, Rohovot, Israel) 5 ug/mL in carbonate/bicarbonate (pH:9.6) to the wells for 24 h. After washes, we added 1% BSA for blocking. Serum samples underwent 1:1000 dilution by PBS and was then added to the well for 24 h. We added anti-mouse IgG (1:1000; Jackson ImmunoResearch, West Grove, PA) and incubated for 2 h. After washes, we added tetramethylbenzidine (TMB) and then H_2_SO_4_ (1 M) to stop the reaction. The results were obtained at OD 450 nm by a TECAN Sunrise ELISA reader (Männedorf, Switzerland).

### 2.4. Determination of Kidney Function

Ethylene diamine tetraacetic acid (EDTA)-anticoagulated blood and urine samples were analyzed using Beckman colter AU480 (Brea, CA). Based on the prior seminal study by Seshan et al. [13] on murine APS nephropathy and another report showing that BUN is more sensitive in detecting murine renal injury than serum creatinine [19], BUN and urine creatinine concentrations were determined. Urine microalbumin concentrations were determined with an ELISA kit (ELK Biotechnology, Denver, CO). We calculated albumin-to-creatinine ratio (ACR) in the urine to determine levels of proteinuria.

### 2.5. Determination of Blood Platelet Count

We determined blood platelet counts, for each mouse, in EDTA-anticoagulated blood samples with the HEMAVET hematology analyzer (Drew Scientific, Miami Lakes, FL).

### 2.6. Administration of the Tyk2 Inhibitor

A Tyk2 inhibitor, BMS-986202 (Bristol-Myers Squibb, NY), was dissolved in the olive oil and given daily to mice at an oral dose of 2 mg/kg from day 35 to 42. In the control APS mice, only the vehicle (olive oil) was given daily from day 35 to 42.

### 2.7. Histological Analysis

Electron microscopy (EM) was performed on retrieved kidney samples. We first fixed kidneys in 4% formaldehyde and 5% glutaraldehyde with 0.1 M sodium cacodylate buffer for 1.5 h and postfixed with 1% osmium tetroxide for 30 min at room temperature. Kidneys were then dehydrated with ethanol before being embedded in LX112 (EMS). Thin sections (80 nm) were stained with uranyl acetate and lead citrate, and we viewed the sections under a HT-7700 transmission electron microscope (Hitachi, Tokyo, Japan). Glomerular injury was evaluated in a semiquantitative way, as described in the literature [13]. Based on the extent of loss of fenestrations, endothelial swelling, and detachment of endothelial cells, we graded the severity of the vascular lesions. Vascular lesions involving less than 25% of the glomeruli were scored as 1, involving 25%–50% of the glomeruli as 2, and involving more than 50% of the glomeruli as 3. In every mouse, we graded at least 5 glomeruli. Scoring was conducted in a treatment-blinded manner. For immunohistochemistry (IHC) staining, kidneys were embedded in paraffin. C3 was detected with specific antibodies (Abcam, Cambridge, UK; 1:2000). Fibrin deposition was detected using a polyclonal antimouse fibrinogen antibody (Biolegend, San Diego, CA; 1:400). The expressions levels of above proteins in the glomeruli were quantified for each mouse, represented by the percent area stained, using the software ImageJ (National Institutes of Health, Bethesda, MD).

### 2.8. Analysis of the IFN Signature in the Kidney

To determine the IFN signature, we first separated the cortex and medulla from the retrieved kidneys. The collected kidney tissues were homogenized with 1 mL Trizol reagent (Sigma, St. Louis, MO) for total RNA extraction. The homogenate was then added to 200 μL of 1-bromo-3-chloropropane (Sigma–Aldrich, China) and thoroughly mixed, followed by centrifugation at 12000 × *g* for 15 min. The supernatants were collected and mixed with 500 μL of isopropanol. We then discarded the supernatants, and the RNA pellet was washed with ethanol. The pellet was dried and resuspended in 50 μL DDW. A total of 2 μg of RNA were mixed with 1 μL of oligo dT (0.5 mM) and DDW, and heated at 70°C for 5 min. Afterwards, we added 1 μL dNTP (10 mM), 5 μL MMLV reverse transcriptase, and DDW for cDNA synthesis and subsequent RT-polymerase chain reaction (PCR) in the StepOne Real-Time PCR System (Applied Biosystems, Waltham, MA). Ten microliters reaction mixture was composed of 5 μL of Fast SYBR Green Master Mix (Applied Biosystems; Thermo Fisher Scientific, UK), 1 μL of 100 μM cDNA, 0.75 μL of primer, and 3.25 μL of DDW. The primers were as follows: Mx1: forward: GAATGGGAAAGTTTTGCCGAGT and reverse: TGATAAACCGTCCACTTAGTCCT; IFN regulatory factor 7 (IRF7) forward: GCGTACCCTGGAAGCATTTC and reverse: GCACAGCGGAAGTTGGTCT; and GAPDH: forward: CGTGTTCCTACCCCCAATGT and reverse: TGTCATCATACTTGGCAGGTTTCT. The obtained data were normalized based on the expression levels of GAPDH. The relative expression level of each target gene was calculated based on the comparative threshold cycle (Ct) in the formula: 2^−*Δ*Ct^, in which *Δ*Ct = sample Ct_target gene_− sample Ct_GAPDH_.

### 2.9. Statistical Analysis

Statistical analyses were performed using GraphPad Prism (version 9.0 for Windows; GraphPad Software). We presented the quantitative data in means and the SDs. One-way ANOVA with post hoc Tukey test was performed for intergroup comparisons (the normal mice, APS mice, and APS mice receiving the Tyk2 inhibitor, BMS-986202). A two-tailed *p* value of <0.05 was recognized as statistically significant.

## 3. Results

### 3.1. The Effect of BMS-986202 on Renal Function

**Figure 1:**
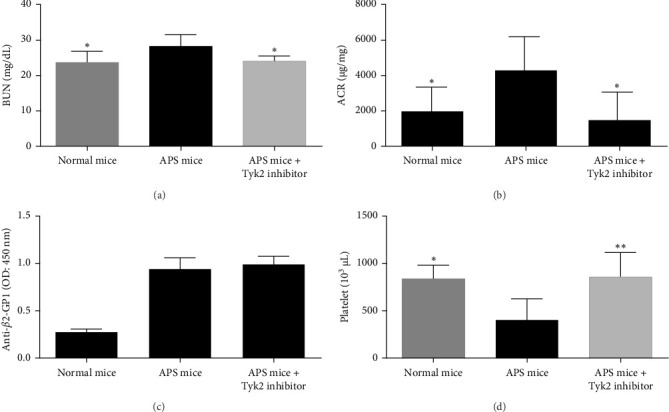
Determination of renal function, serum levels of IgG anti-*β*2-GPI antibody, and blood platelet count in mice. Comparisons of (A) BUN, (B) the amount of microalbuminuria, (C) serum levels of IgG anti-*β*2-GPI antibody, and (D) blood platelet count between groups of mice. The results were presented in means ± SD from six mice/group. *⁣*^*∗*^*p* < 0.05, *⁣*^*∗∗*^*p* < 0.01 versus the APS mice. *β*2-GPI, *β*2-glycoprotein I; ACR, albumin-to-creatinine ratio; APS, antiphospholipid antibody syndrome; BUN, blood urea nitrogen; SD, standard deviation; Tyk2, tyrosine kinase 2.

As shown in Figure 1A,B, we observed elevated levels of BUN and albuminuria in APS mice when compared with the normal mice. BMS-986202 alleviated such changes.

### 3.2. The Effect of BMS-986202 on the Production of Anti-*β*2-GPI Antibodies

As shown in Figure 1C, production of serum anti-*β*2-GPI antibodies was elevated after *β*2-GPI immunization. BMS-986202, however, did not affect the serum levels of anti-*β*2-GPI antibodies.

### 3.3. The Effect of BMS-986202 on Platelet Count

As shown in Figure 1D, a decrease in blood platelet count was noted in the APS mice, which was reversed after administration of BMS-986202.

### 3.4. Histological Effects of BMS-986202 on APS Nephropathy

**Figure 2:**
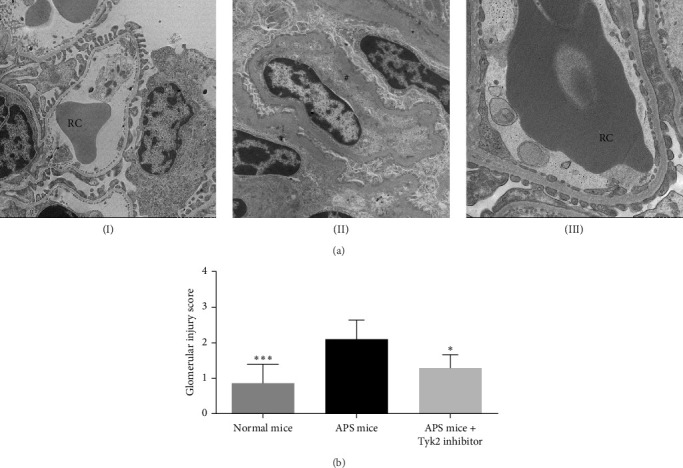
Glomerular vascular injury in mice with APS nephropathy. (A) Representative transmission electron micrograph of vascular lesions in the kidneys (magnification ×2500) of (I) normal mice, (II) APS mice, and (III) APS mice receiving BMS-986202. Swelling of glomerular capillary endothelial cells (*⁣*^*∗*^) and loss of fenestrations (#) were observed in glomeruli from APS mice, which was reversed after administration of BMS-986202. (B) Comparisons of glomerular injury scores between groups of mice. The results were presented in means ± SD from six mice per group. *⁣*^*∗*^*p* < 0.05; *⁣*^*∗∗∗*^*p* < 0.001 versus the APS mice. APS, antiphospholipid antibody syndrome; RC, red blood cell; SD, standard deviation; Tyk2, tyrosine kinase 2.

**Figure 3:**
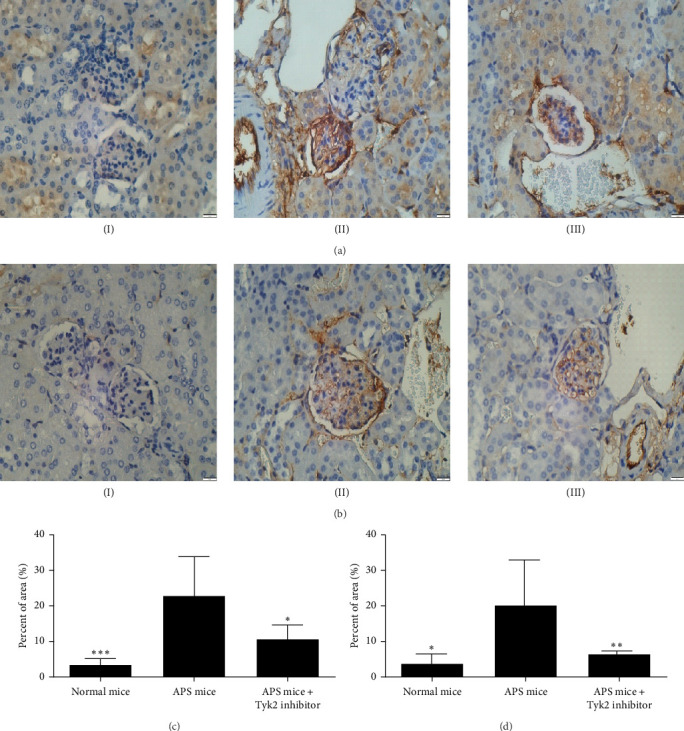
Mouse glomerular fibrin and C3 deposition determined by immunohistochemistry (IHC). Representative images of glomerular (A) fibrin and (B) C3 deposition in the kidneys (magnification 400x) of (I) normal mice, (II) APS mice, and (III) APS mice receiving BMS-986202. Comparisons of expression of (C) fibrin and (D) C3 between groups of mice as determined by ImageJ. The results were presented in mean ± SD from six mice per group. *⁣*^*∗*^*p* < 0.05; *⁣*^*∗∗*^*p* < 0.01; *⁣*^*∗∗∗*^*p* < 0.001 versus the APS mice. APS, antiphospholipid antibody syndrome; SD, standard deviation; Tyk2, tyrosine kinase 2.

We further examined the ultrastructural effects of BMS-986202 on APS nephropathy. As shown in Figure 2, immunization of *β*2-GPI in mice led to more severe vascular lesions in glomeruli when compared with the normal mice. Administration of BMS-986202 reversed such abnormalities. In addition, as shown in Figure 3, glomerular deposition of fibrin and C3 were greater in APS mice when compared with the normal mice and such greater deposits were reversed by BMS-986202.

### 3.5. The Effect of BMS-986202 on the IFN Signature in the Kidney

**Figure 4:**
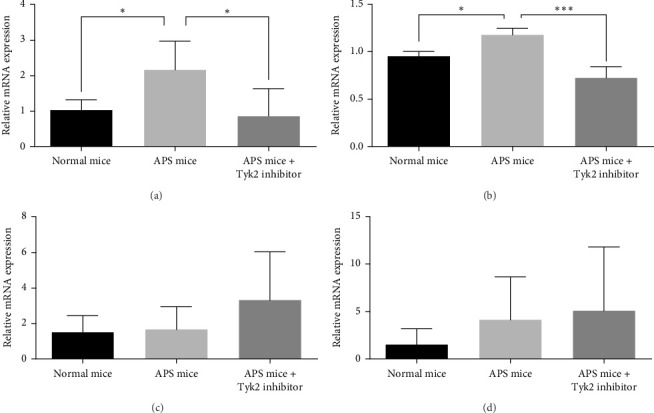
The IFN signature in the mouse kidneys as determined by real-time PCR. The expression levels of IRF7 and Mx1 in the (A, B) cortex and (C, D) medulla of kidneys in mice. The bar graph represents the mean ± SD of six mice per group. *⁣*^*∗*^*p* < 0.05; *⁣*^*∗∗∗*^*p* < 0.001 versus the APS mice. APS, antiphospholipid antibody syndrome; IFN, interferon; IRF7, IFN regulatory factor 7; PCR, polymerase chain reaction; SD, standard deviation; Tyk2, tyrosine kinase 2.

As shown in Figure 4, we found that in the renal cortex, but not in the medulla of murine kidneys, mRNA expression levels of those genes downstream of type I IFN signaling (IRF7 and Mx1) were upregulated. Such an upregulation was reversed after administration of BMS-986202.

## 4. Discussion

This is the first study on the therapeutic potential of Tyk2 inhibitors in a murine model of APS nephropathy. We found that inhibiting Tyk2 had suppressed manifestations of APS nephropathy. This effect is likely mediated through suppressing the type I IFN response in the kidney.

APS nephropathy is prevalent in APS patients but difficult to treat, with no established treatment. Here, we have demonstrated in mice that *β*2-GPI immunization produced anti-*β*2-GPI antibodies, which in turn affected the kidney. Consistent with the previous report by Seshan et al. [13], we showed histological damage of murine kidneys, including vascular lesions by EM and fibrin and C3 deposition at the glomeruli. Furthermore, BUN and microalbuminuria levels were higher in APS mice, providing biochemical evidence on deteriorated renal function. These findings support our murine model being appropriate, at least in part, to represent APS nephropathy.

The pathogenesis of APS nephropathy is elusive. Tissue factor, complement pathway, and mTOR activation are implicated [10, 13]. In recent decades, the type I IFN response has been shown to play a key role in the generation of systemic autoimmune diseases such as SLE and Sjogren's syndrome [20, 21]. In addition, a prior study reported that IFN-*α* augments inflammatory responses at the atherosclerotic plaque, partly through the upregulation of Toll-like receptor 4 (TLR4) expression in myeloid dendritic cells [22]. Consistent with these findings, the type I IFN score was reported to be higher in the blood of APS patients and further correlated with the presence of anti-*β*2-GPI antibodies [23]. Our murine model also demonstrated that the IFN signature was upregulated in the kidneys of APS mice. Interestingly, type I IFN therapy has been reported, in patients with multiple sclerosis, to be associated with thrombotic microangiopathy (a major pathological finding in APS nephropathy) and type I IFN signature in their renal biopsies [24]. Thrombotic microangiopathy after hematopoietic stem cell transplantation is also associated with an upregulated type I IFN response [25]. Anifrolumab, a human monoclonal antibody for type I IFN receptor, is currently an approved treatment option for SLE [26]. Further studies on the type I IFN response in patients with APS nephropathy should be conducted.

Upon ligand binding, the JAK family is capable of phosphorylating the cytokine receptor, which then recruits signal transducer and activator of transcription (STAT) to be further phosphorylated. Activated STAT translocates to the nucleus. There, it acts as a transcription factor to regulate cellular function. The JAK family includes JAK1, JAK2, JAK3, and Tyk2, and their inhibition is known to be therapeutic for many autoimmune and hematological diseases [27]. Tyk2 is indispensable for signaling of several pro-inflammatory cytokines, including IL-12 and IL-23 as well as IFN-*α* [18]. BMS-986202 is a novel chemical compound chemically analogous to deucravacitinib (BMS-986165), which is approved for treating psoriasis [16]. BMS-986202 binds to the JH2 domain of Tyk2 and thereby, inhibits its downstream signaling. In accordance with that, we found that BMS-986202 could suppress the IFN signature in the kidney of APS mice. Nevertheless, the production of anti-*β*2-GPI antibodies and *T* helper response in murine spleen cells were not affected (data not shown) despite previous studies showing that type I IFN could enhance B and T cells [28–30]. This may be related to the growth-inhibitory effect of type I IFN on immune cells [31]. Furthermore, the pathological change and biochemical abnormalities of APS nephropathy were reversed after BMS-986202 administration, although, we did not evaluate the real function (glomerular filtration rate) by the more accurate methods such as clearance of inulin, iohexol, or radioactive isotopes. Our results highlight the pathogenic role of type I IFN response and likely provide a novel treatment option for APS nephropathy. BMS-986202, without cellular toxicity reported in a preprint [32], is a potential therapeutic candidate.

## 5. Conclusions

The pathogenesis and appropriate treatment for APS nephropathy are largely unknown. We have demonstrated the potential role of type I IFN in the pathogenesis of APS nephropathy. In addition, the therapeutic efficacy of BMS-986202, a novel Tyk2 inhibitor, has been demonstrated in a murine model of APS nephropathy. Further human studies are needed.

## Data Availability

The data that support the findings of this study are available on request from the corresponding author.

## Nomenclature

ACR: albumin-to-creatinine ratio
APS: antiphospholipid antibody syndrome
aPL: antiphospholipid antibodies
*β*2-GPI: *β*2-glycoprotein I
BUN: blood urea nitrogen
EDTA: ethylene diamine tetraacetic acid
EM: electron microscopy
IFN: interferon
IL: interleukin
IHC: immunohistochemistry
IRF7: IFN regulatory factor 7
JAK: Janus kinase
JH1: JAK homology 1
JH2: JAK homology 2
STAT: signal transducer and activator of transcription
SLE: systemic lupus erythematosus
TLR4: Toll-like receptor 4
TMB: tetramethylbenzidine
Tyk2: tyrosine kinase 2
